# Influence of hyperbaric oxygen on biomechanics and structural bone matrix in type 1 diabetes mellitus rats

**DOI:** 10.1371/journal.pone.0191694

**Published:** 2018-02-16

**Authors:** Pedro Henrique Justino Oliveira Limirio, Huberth Alexandre da Rocha Junior, Richarlisson Borges de Morais, Karen Renata Nakamura Hiraki, Ana Paula Coelho Balbi, Priscilla Barbosa Ferreira Soares, Paula Dechichi

**Affiliations:** 1 Department of Histology, Federal University of Uberlândia, Uberlândia, Minas Gerais, Brazil; 2 Faculty of Nursing, Federal University of Mato Grosso, Cuiabá, Mato Grosso, Brazil; 3 Department of Physiology, Federal University of Uberlândia, Uberlândia, Minas Gerais, Brazil; 4 Department of Periodontology and Implantology, Federal University of Uberlândia, Uberlândia, Minas Gerais, Brazil; Rensselaer Polytechnic Institute, UNITED STATES

## Abstract

**Background:**

The aim of this study was to evaluate the biomechanics and structural bone matrix in diabetic rats subjected to hyperbaric oxygen therapy (HBO).

**Methods:**

Twenty-four male rats were divided into the following groups: Control; Control + HBO; Diabetic, and Diabetic + HBO. Diabetes was induced with streptozotocin (STZ) in the diabetic Groups. After 30 days, HBO was performed every 48h in HBO groups and all animals were euthanized 60 days after diabetic induction. The femur was submitted to a biomechanical (maximum strength, energy-to-failure and stiffness) and Attenuated Total Reflectance Fourier transform infrared (ATR-FTIR) analyses (crosslink ratio, crystallinity index, matrix-to-mineral ratio: Amide I + II/Hydroxyapatite (M:MI) and Amide III + Collagen/HA (M:MIII)).

**Results:**

In biomechanical analysis, diabetic animals showed lower values of maximum strength, energy and stiffness than non-diabetic animals. However, structural strength and stiffness were increased in groups with HBO compared with non-HBO. ATR-FTIR analysis showed decreased collagen maturity in the ratio of crosslink peaks in diabetic compared with the other groups. The bone from the diabetic groups showed decreased crystallinity compared with non-diabetic groups. M:MI showed no statistical difference between groups. However, M:MIII showed an increased matrix mineral ratio in diabetic+HBO and control+HBO compared with control and diabetic groups. Correlations between mechanical and ATR-FTIR analyses showed significant positive correlation between collagen maturity and stiffness.

**Conclusions:**

Diabetes decreased collagen maturation and the mineral deposition process, thus reducing biomechanical properties. Moreover, the study showed that HBO improved crosslink maturation and increased maximum strength and stiffness in the femur of T1DM animals.

## Introduction

Type 1 diabetes mellitus (T1DM) is a metabolic disorder characterized by chronic hyperglycemia that affects various human body systems [1]. Children and adolescents with T1DM are at risk for presenting a decrease in bone mass during the process of bone remodeling. This may minimize the attainment of peak bone mass and increase the fracture risk and/or osteoporosis in adulthood [2]. Some studies have suggested that T1DM negatively changed the collagen in bone matrix and reduced the necessary maximum bone fracture strength [3, 4].

T1DM has been associated with cellular and molecular changes that result in bone matrix alterations [5]. The chronic hyperglycemia has deleterious effects on structural collagen protein and this may change the biomechanical behavior of bone tissue [6]. Studies have revealed that diabetes decreased total collagen content and deteriorated pyridinoline crosslinking in fracture calluses [6, 7]. In addition, T1DM can affect bone formation and resorption, leading to several metabolic irregularities in calcium-phosphate and acid-base balances [8]. Indeed, T1DM exhibits disproportionately high fracture risk with reduced bone mass, which leads to speculation about diabetic bone having reduced maximum strength [9] and stiffness [10]. The deleterious effects of diabetes on bone response to mechanical stimuli have been shown in the literature, so it is important to investigate alternative therapy to improve bone quality [6].

Hyperbaric oxygen therapy (HBO) has been used to treat cases with impairment of repair for decades [11]. It consists of intermittent inhalation of 100% oxygen under a pressure higher than 1.5 atmospheres absolute [12]. Studies have suggested that HBO activates several mechanisms that contribute to repair [13], including increased collagen synthesis [14] and stimulation of the bone repair process [15]. Moreover, some studies have suggested that HBO induces enzymatic crosslinking, which contributes to the bone mineralization process [16]. This procedure stimulates the incorporation of mineral crystals into collagen crosslinks and increases the maximum breaking strength values in rat femurs [17]. However, the HBO effect on diabetic bone in a rat model has not been studied.

In this study, it was hypothesized that HBO would improve bone biomechanical properties (maximum strength, energy and stiffness), and the collagen and crystalline hydroxyapatite content in diabetic femurs in an animal model. Therefore, the aim of the present study was to evaluate T1DM and HBO effect on the rat femur, using biomechanics and Attenuated Total Reflectance Fourier Transform Infrared Spectroscopy (ATR—FTIR) analyses.

## Material and methods

### Experimental procedure

This study was approved by the Science and Ethics Committee of the Federal University of Uberlândia (026/14), Brazil, and was conducted in accordance with the Brazilian College for Animal Experimentation (COBEA) guidelines. The sample consisted of 24 male Wistar rats (*Rattus norvegicus*), weighing 240 to 280g (8 weeks of age). The animals were kept in cages, in a 12h:12h light-dark cycle, and controlled temperature conditions (22 ± 2°C), with standard food and water *ad libitum*. The animals were randomly divided into four groups (n = 6), as follows: Control; Control + HBO; Diabetic and Diabetic + HBO. The oxygen therapy was performed every 48h and started 30 days after streptozotocin (STZ) (Sigma Aldrich, St Louis, MO, USA) induced T1DM. All animals were euthanized 60 days after diabetic induction.

The T1DM induction protocol began by keeping the rats fasting for 24h. After this time and before T1DM induction, the mean blood glucose level of the animals was 100 mg/dL. Anesthesia was performed via the intraperitoneal pathway using 7mg/Kg xylazine 2% muscle relaxant, and 100mg/Kg ketamine hydrochloride 10% anesthetic and analgesic. Then, a single dose of STZ was administered intravenously through a penile vein puncture at a dose of 65 mg/kg body weight, diluted in citrate buffer. Hyperglycemia was confirmed by a glucometer (Accu Check Active, Roche, Jaguaré, SP, Brazil) after 24 hours; one week; 15 days, and 30 days after induction, by collecting a drop of blood from each animal’s tail. Animals that maintained blood glucose levels higher than 200 mg/dL were considered diabetic. The animals that did not reach the glycemic target were excluded from the study.

HBO was performed 30 days after STZ-induction in control+HBO and diabetic+HBO groups, and was repeated every 48h, so the animals received 15 HBO sessions. The treatment was realized in a cylindrical pressure chamber Ecobar 400 (Ecotec Equipamentos e Sistemas Ltda®, Mogi das Cruzes, SP, Brasil) at 2.5 ATA for 90 min. The animals were euthanized 60 days after diabetes induction by intraperitoneal injection with sodium thiopental and lidocaine, followed by cervical dislocation, in compliance with the principles of the Universal Declaration on Animal Welfare.

Both femurs were removed by disarticulation and immediately placed in gauze impregnated with physiological saline solution and then kept frozen in a freezer (-20°C). Twenty-four hours before the mechanical test, the femurs were defrosted and placed in phosphate buffered saline until they were analyzed.

### Biomechanical and attenuated total reflectance Fourier Transform Infrared Spectroscopy (ATR—FTIR) analyses

Each femur was analyzed in a three-point bending test until failure, using universal-testing machine (EMIC DL 2000, EMIC Equipamentos e Sistemas de Ensaio Ltda, Sao José dos Pinhais, Brazil). Each specimen was placed horizontally on the two holding fixtures (16 mm) in the machine, the upper device load was applied in middle of the diaphysis at a loading rate of 1.0 mm/min. Load, displacement data were recorded, and subsequently, load vs. displacement curves were plotted. Evaluations were derived from data with maximum strength (N), energy-to-failure (mJ) and stiffness values (N/mm) and calculated as the slope of the initial linear uploading portion of the curves. Femurs fractured after the mechanical test were maintained in phosphate buffered saline until the attenuated total reflectance Fourier transform infrared spectroscopy (ATR—FTIR) analysis.

**Fig 1:**
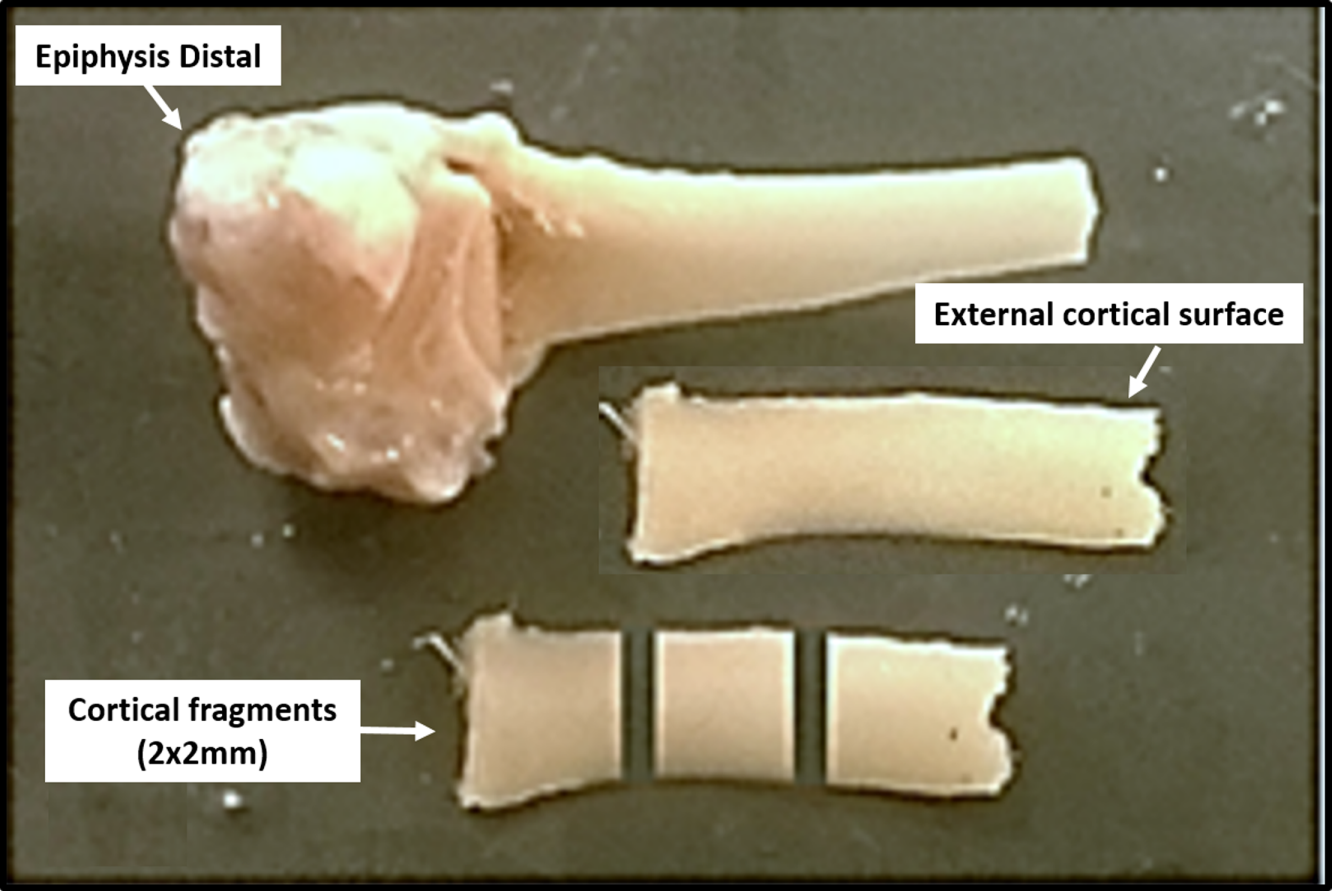
Cortical segments of the femur obtained to perform analysis by the ATR-FTIR.

After the three-point bending test, the proximal diaphysis was sectioned with a diamond disk under constant irrigation to obtain three fragments measuring 2x2 mm, with 2 mm thick (Fig 1). The mean values of three spectrums in each femur were obtained on the external cortical surface. The bone fragment was placed against the diamond crystal of the ATR-FTIR unit and pressed with a force gauge at a constant pressure to facilitate contact. Data were recorded and analyzed with OPUS 6.5 software (Bruker, Ettlingen, Germany). The bone composition was analyzed using Fourier Transform Infrared Spectroscopy (ATR-FTIR, Vertex 70 –Bruker, Ettlingen, Germany) equipped with an accessory that allowed spectrum acquisitions in the Attenuated Reflectance (ATR) mode. The ATR spectrums were recorded in the range of 400–4,000 cm^−1^ at a 4 cm^−1^ resolution. Vector normalization and baseline correction were performed in all spectrums and these were considered absorbance height ratios.

**Fig 2:**
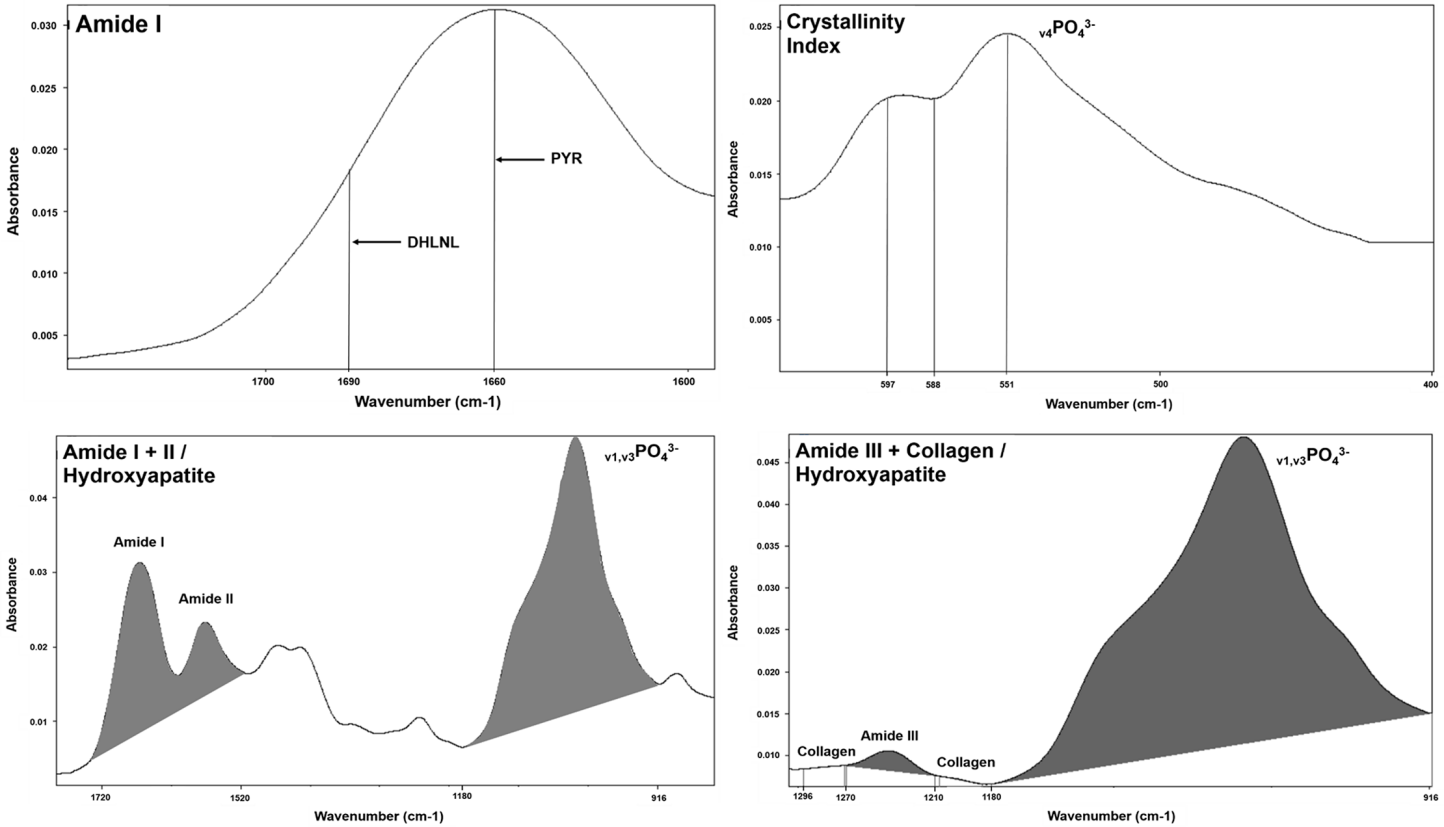
Parameters analyzed by means of ATR-FTIR spectrums using the program OPUS 6.5. Amide I band (Collagen ratio between the mature pyridinoline crosslink peaks (PYR)– 1660 cm-1 and immature crosslinking dihydroxinorleucina (DHLNL) - 1690 cm-1); Crystallinity Index (The intensity ratio of peaks 551 and 597 cm-1 for 588 cm-1); Matrix-to-mineral ratio: Amide I + II/Hydroxyapatite (HA) (The ratio between integrated areas of amide I + II (1520–1720 cm-1) for HA (916–1180 cm-1)) and Amide III + Collagen/HA (The ratio between integrated areas of amide III (1210–1270 cm-1) with two collagen bands (1269–1296 cm-1 and 1180–1213 cm-1) for HA (916–1180 cm-1).

The ATR-FTIR spectrums were further analyzed by calculating the following parameters: Amide I band (Collagen ratio between the mature pyridinoline crosslink peaks (PYR)– 1660 cm^-1^ and immature crosslinking dihydroxynorleucine (DHLNL) - 1690 cm^-1^); Crystallinity Index (The intensity ratio of peaks 551 and 597 cm^-1^ for 588 cm^-1^); Matrix-to-mineral ratio: Amide I + II/Hydroxyapatite (HA) (M:MI) (The ratio between integrated areas of amide I + II (1520–1720 cm^-1^) for HA (916–1180 cm^-1^)) and Amide III + Collagen/HA (M:MIII) (The ratio between integrated areas of amide III (1210–1270 cm^-1^) with two collagen bands (1269–1296 cm^-1^ and 1180–1213 cm^-1^) for HA (916–1180 cm^-1^) [18, 19] (Fig 2).

### Statistical analysis

Analysis was performed using statistical software Sigma Plot 13.1^®^ (Systat Software Inc, San Jose, CA, USA). The results obtained were submitted to the Kolmogorov-Smirnov normality test and Two-Way Anova followed by the Tukey test. Correlation between biomechanics and ATR-FTIR analysis was measured by Pearson’s correlation. Differences were considered statistically significant when α<0.05.

## Results

Throughout the experimental procedure, the animals of diabetic and diabetic+HBO groups maintained hyperglycemia (glucose levels above 200 mg/dl), weight reduction, polyphagia, polydipsia and polyuria, observed from the increase in feed and water intake, and urinary excretion.

### Mechanical analysis

**Fig 3:**
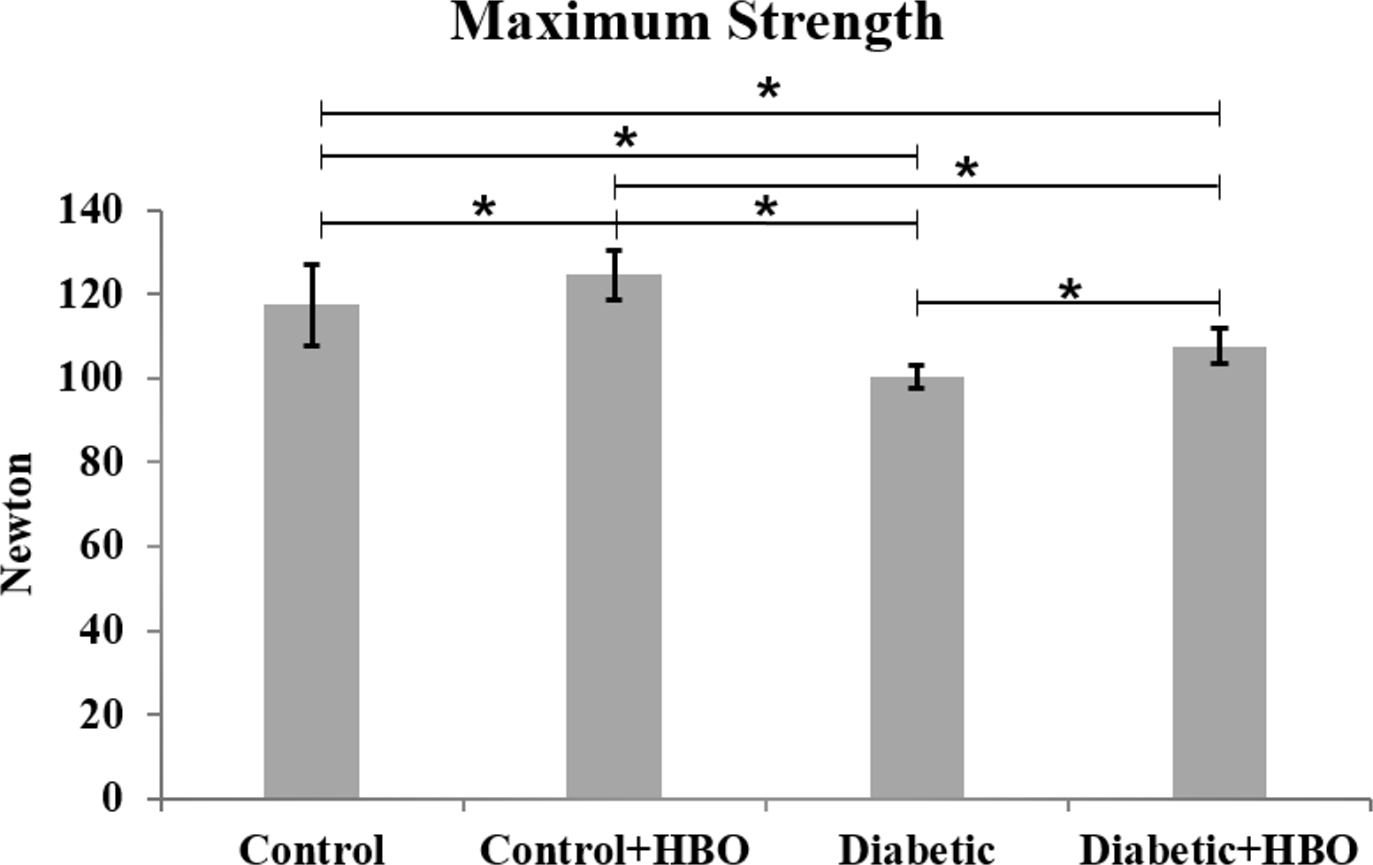
Maximum strength of biomechanical analyses in different groups (*p<0.05).

**Fig 4:**
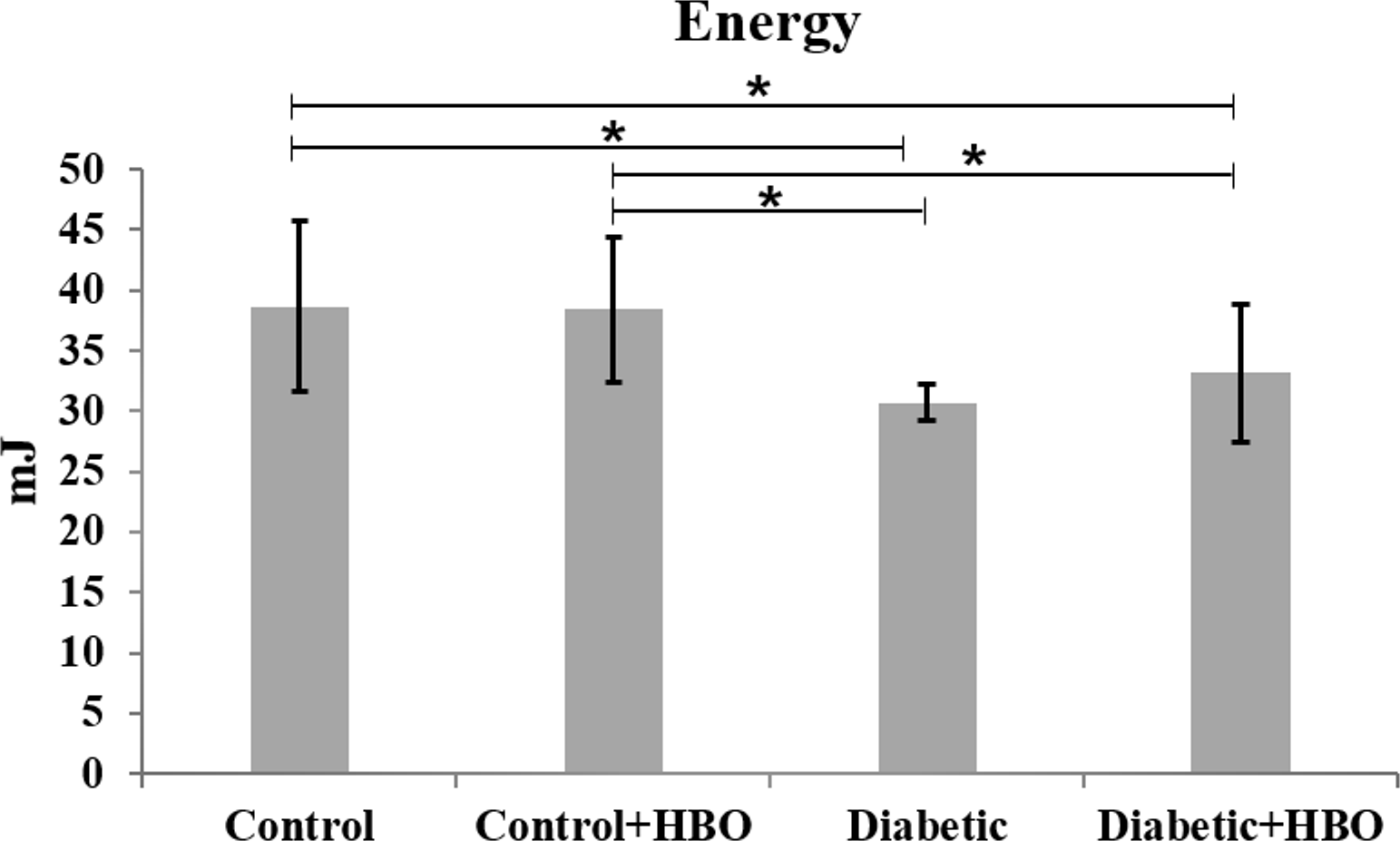
Energy of biomechanical analyses in different groups (*p<0.05).

**Fig 5:**
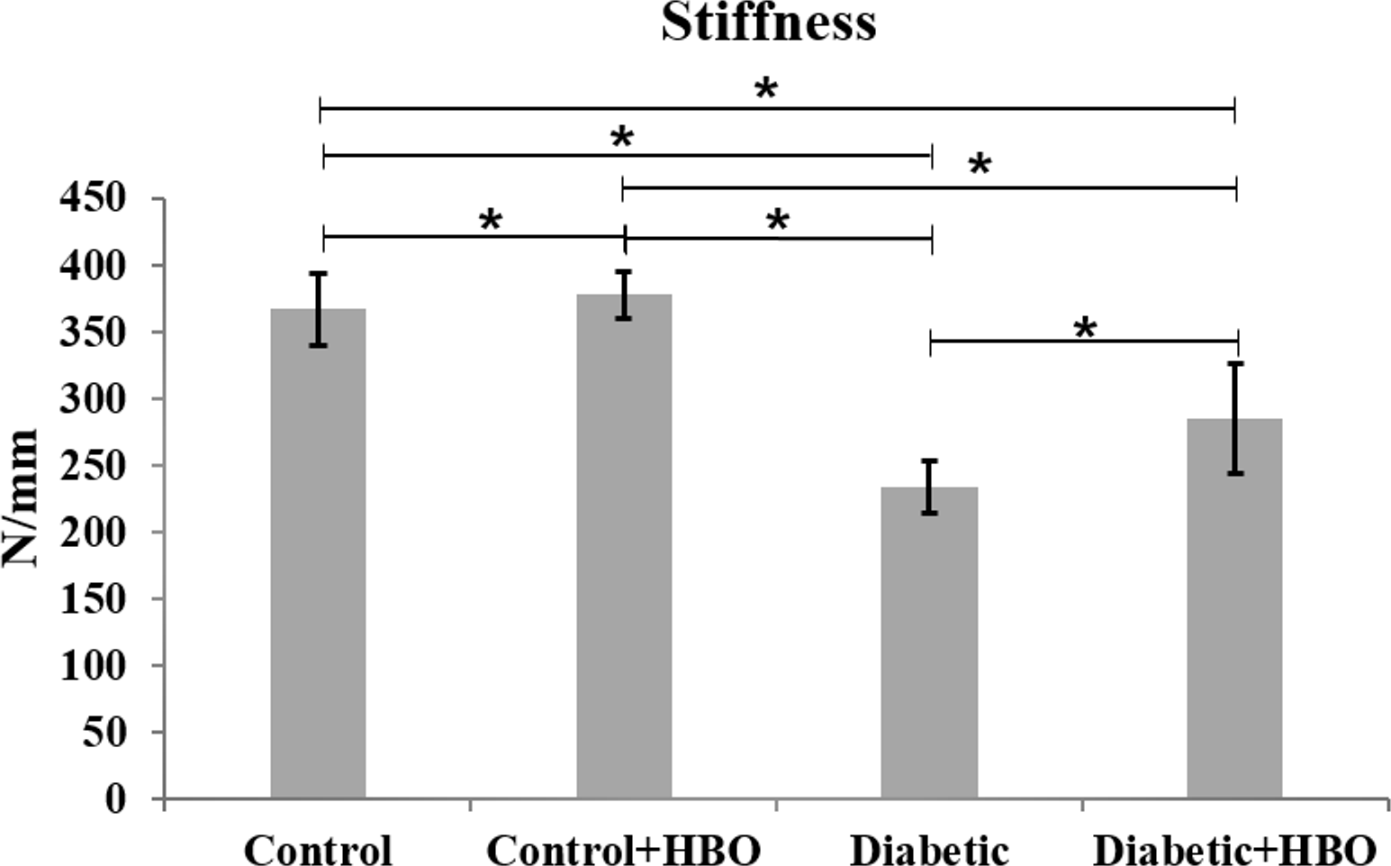
Stiffness of biomechanical analyses in different groups (*p<0.05).

In the mechanical analysis, diabetic groups showed lower values of maximum strength ((diabetic (100.5±5.6) and diabetic+HBO (107.0±8.8) vs control (117.7 ± 11.3) and control+HBO (124.2 ± 11.6)) stiffness ((diabetic (233.4 ± 33.1) and diabetic+HBO (277.9 ± 43.0) vs control (366.5 ± 37.6) and control+HBO (377.7 ± 26.0)) and energy ((diabetic (30.6 ± 3.6) and diabetic+HBO (33.0 ± 8.6) vs control (38.8 ± 8.2) and control+HBO (38.3 ± 8.3)) than non-diabetic animals (p<0.007). However, there were increases in the maximum strength and stiffness values in HBO groups (control+HBO and diabetic+HBO) compared with non-HBO groups (control and diabetic) (p<0.042) (Figs 3–5).

### ATR-FTIR analysis

**Fig 6:**
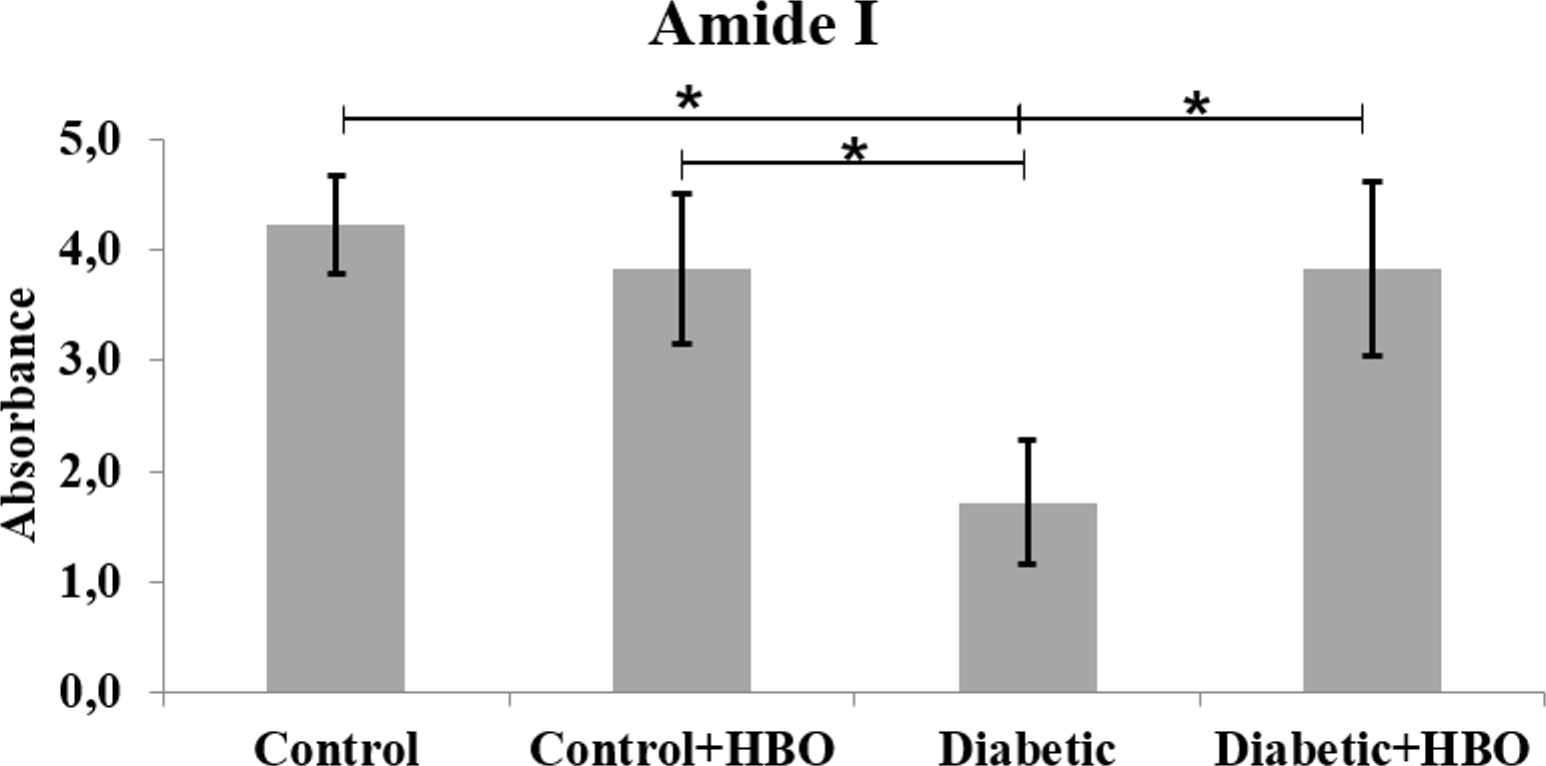
Amide I of ATR-FTIR analyses in different groups (*p<0.05).

**Fig 7:**
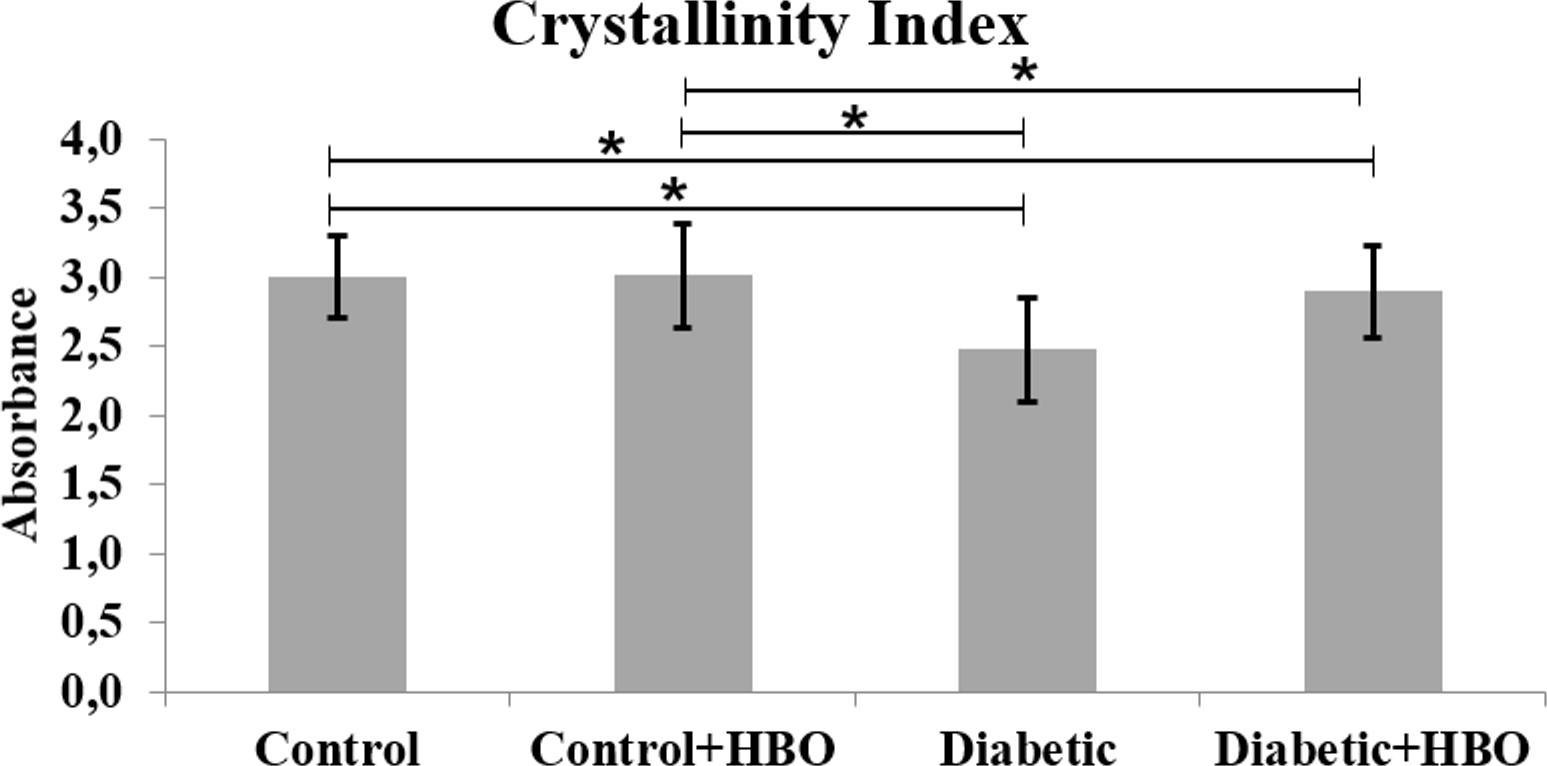
Crystallinity index of ATR-FTIR analyses in different groups (*p<0.05).

**Fig 8:**
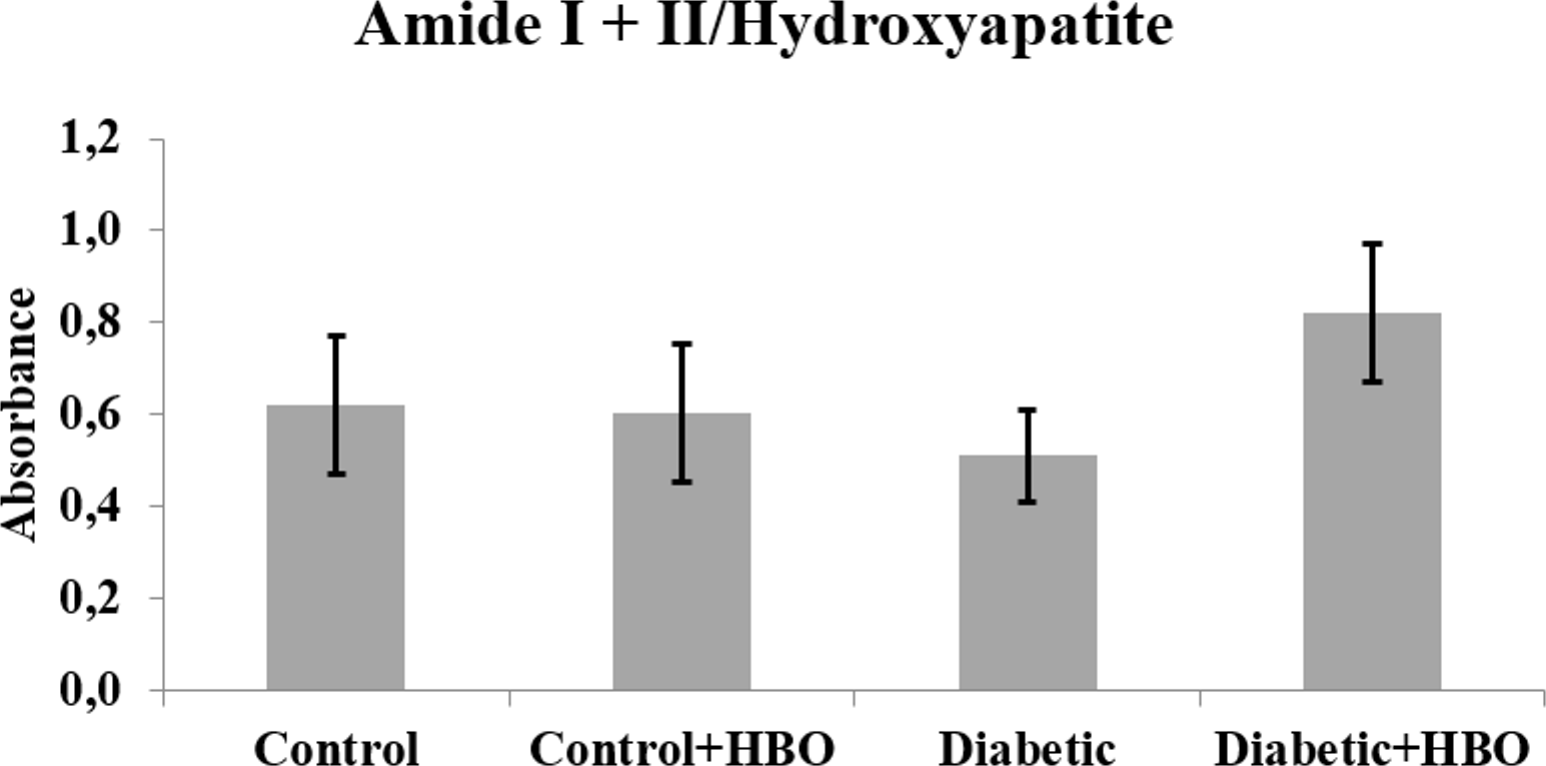
Matrix: Mineral ratio (Amide I+II/Hydroxyapatite) of ATR-FTIR analyses in different groups (*p<0.05).

**Fig 9:**
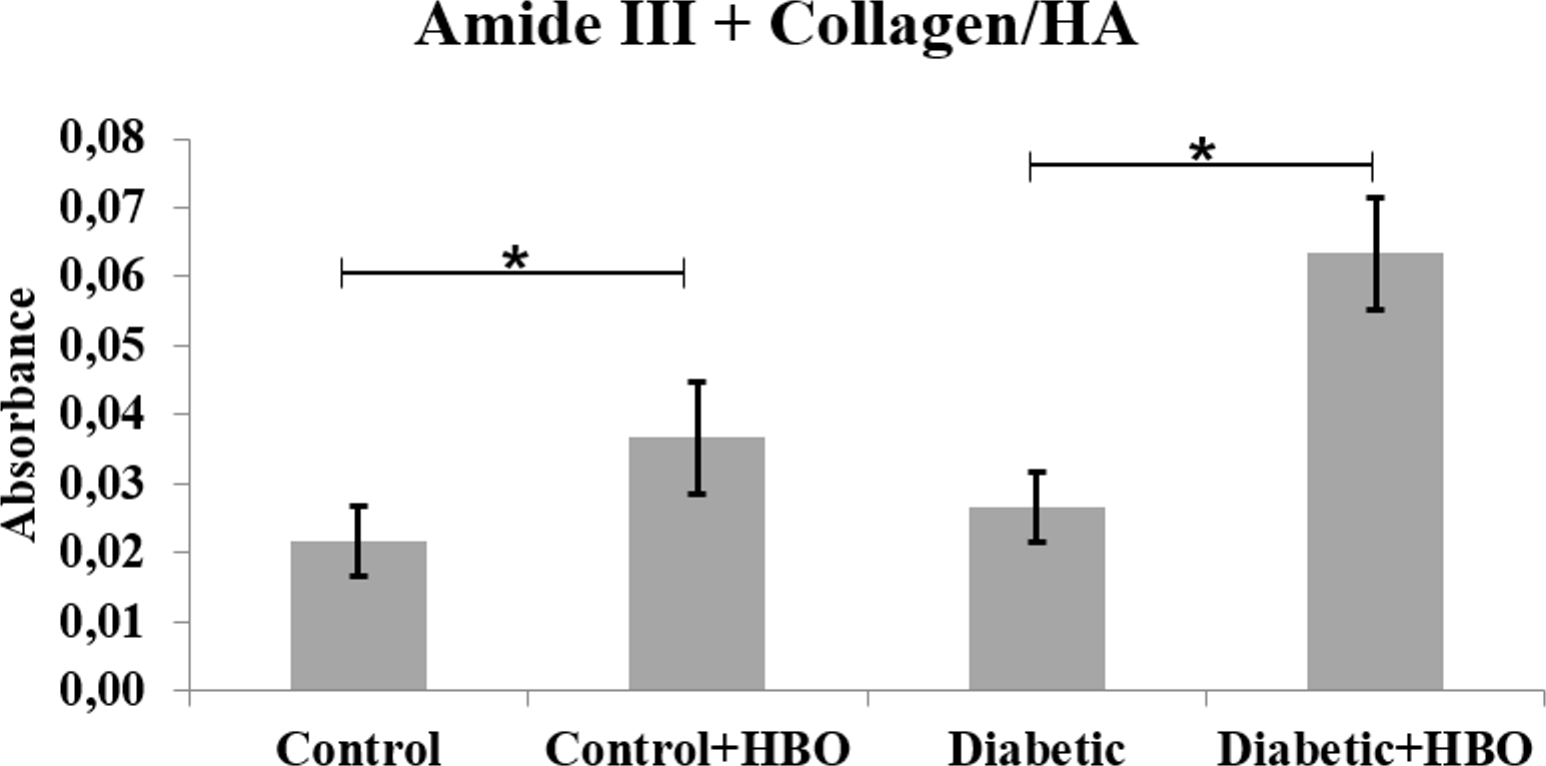
Matrix: Mineral ratio (Amide III+Collagen/Hydroxyapatite) of ATR-FTIR analyses in different groups (*p<0.05).

In the spectrums, main bands, characteristic of bone components were observed. The collagen maturity analysis showed a decreased ratio of crosslink peaks in diabetic (1.72±1.12) compared with the other groups (control (4.23±0.88), control+HBO (3.83±1.37) and diabetic+HBO (3.83±1.58) (p = 0.003) (Fig 6). The bone from the non-diabetic groups presented increased crystallinity compared with those from the diabetic groups (control (3.01±0.30) and control+HBO (3.02±0.38) vs diabetic (2.48±0.38) and diabetic+HBO (2.90±0.33)) (p<0.034) (Fig 7). Matrix-to-mineral ratio evaluation of M:MI showed no statistical difference between groups control (0.62±0.38), control+HBO (0.60±0.40), diabetic (0.51±0.28) and diabetic+HBO (0.82±0.37) (p>0.278) (Fig 8). For the parameter, M:MIII there was an increase in matrix mineral ratio in diabetic+HBO (0.06±0.04) and control+HBO (0.04±0.04) compared with diabetic (0.03±0.01) and control (0.02±0.02), respectively (p = 0.035) (Fig 9). Correlations between mechanical tests and ATR-FTIR analyses showed significant positive correlation between collagen maturity and stiffness (r = 0.56, p = 0.02).

## Discussion

The present study hypothesized that HBO would improve the bone matrix composition and mechanical properties in diabetic rats. In fact, our results showed that HBO minimized the deleterious effect of T1DM on collagen maturation and increased maximum strength and stiffness in diabetic rat femurs.

T1DM is an autoimmune insulin-dependent disease characterized by remarkable reduction in insulin production and chronic hyperglycemia, and accounts for approximately 10% of all diabetes cases. T1DM is associated with younger people [20], and due to longer exposure time to the disease, it generally has more serious repercussions on tissues, compared with diabetes mellitus type 2 [21].

The most common methods for establishing T1DM rodent models [22] is by means of a high-dose injection with STZ, which rapidly destroys pancreatic β cells and results in typical human T1DM symptoms [23]. Some studies have shown that STZ-induction decreased bone formation, deteriorated bone architecture, and compromised skeletal health, quality and strength, which revealed similarity to the bone phenotype of T1DM in human patients [23, 24].

The decreased maximum biomechanical strength, energy and stiffness in diabetic groups suggest that T1DM increased the fracture risk, which may be due to the structural changes in bone. Consistent with these finding, studies have shown that diabetes decreased bone strength, energy absorption [9, 25], and mineral content in diabetic rats [8].

Bone fracture resistance depends on several bone characteristics, and has been described as a multiple-scale process, the scale of which has a level within the structural hierarchy [6]. It is likely that T1DM, in some way, compromises the bone hierarchical structure, reducing its resistance, which could explain our results. The macroscopic structure (size and shape), architecture (cortical and cancellous components) and the bone substance (organic and inorganic components) are also influenced by T1DM [25].

Bone is a two-phase composite material in which the mineral phase provides stiffness and the collagen provides strength and the post-yield property of ductility [17]. Bone matrix development starts when the collagen fibrils appear and follows a process of enzymatically induced cross-linking that stabilizes the fibrils [17]. The collagen fibrils serve as scaffolds on which nucleation and growth of the mineral crystals will take place. These two processes are intimately correlated as shown by the similar trend between the crystallinity and the collagen crosslink ratio pattern across the osteons [26].

In the present study, the collagen maturity analysis showed decreased enzymatic crosslink peak ratios in diabetic when compared with the other groups. This decrease suggested that there was a higher proportion of immature crosslinks compared with the mature crosslinks in diabetic animals. Indeed, either an increase in immature DHLNL crosslinks (intrafibrillar) or a reduction in mature PYR crosslinks (interfibrillar) could disrupt the mature crosslink integrity, leading to decreased energy and premature bone failure [27]. The collagen crosslink ratio indicates the state of maturity of the crosslinking network in the bone collagen fibrils, which is important for the structural and mechanical properties of bone [26].

The degree in collagen crosslink formation is regulated by the extent of glycation [17]. Experimental studies have shown that advanced glycation end products (AGEs) are formed when free-floating sugars interact with exposed amino acid residues on collagen, resulting in a reversible Amadori intermediate that ultimately undergoes oxidation to form irreversible AGEs [28, 29]. These compounds accumulate and affect cross-links within type 1 collagen [29, 30]. The AGEs impair immature and mature crosslinks in the collagen matrix contributing to bone fragility [30, 31], which could be associated with the results of the present study.

Diabetic animals submitted to HBO showed an increased PYR/DHLNL crosslink ratio. Intermolecular collagen crosslinking is important for development of the underlying matrices that are essential for initial mineral formation and crystal growth [26]. According to a previous study, a specific PYR induces the type of enzymatic crosslinking pattern that influences bone matrix mineralization, increasing bone maximum strength and stiffness [17], as shown in our study. However, the mechanism of how HBO changes the crosslinks in diabetes is unknown [32].

In the present study, crystallinity decreased in diabetic compared with non-diabetic animals. This result suggested that TIDM increased the presence of large HA crystals and decreased the surface area in collagen fibrils [33]. Boyar et al showed that crystallite sizes were changed in the bone mineral matrix of diabetic rats [8]. The highly ordered location and orientation of very small crystals within the collagen fibrils contribute to the bone rigidity and strength. In addition, their small size allows an acceptable range of flexibility without fracture or disruption of the bone substance [34]. Recent studies have suggested that increased bone mineral particle size was associated with increased bone fragility [26].

The positive correlation between collagen maturity and stiffness showed that both parameters decreased in animals with TIDM. This could be due changes in the enzymatic process that induces fibril stabilization by collagen crosslinking [17], leading to deterioration in mineralization [35] and decreased bone stiffness [6]. Recently, some studies on diabetic bone showed that hyperglycemia affected the type I collagen and compromised the mineralization process [17, 26, 27].

M:MI showed no statistical difference between the groups. This suggested that TIDM reduced collagen maturity and crystallinity in the same proportions, thus without change in the ratio between the organic and inorganic matrix. Some studies in humans and animals have shown that diabetes impaired bone metabolism, leading to decreased bone mass [4]. However, M:MIII showed that the matrix mineral ratio increased in control+HBO and diabetic+HBO compared with control and diabetic groups. Our results suggested that HBO increased intermolecular interactions (by hydrogen bonds) in the collagen, followed by induced cross-linking that stabilized the fibrils [36], which explained the increase maximum strength and stiffness in HBO groups.

Although there was no statistical difference between groups, DH showed higher values in M:MI and M:MIII. It could be that the effect of T1DM [26] and HBO [36] on collagen crosslinks increased the interaction with fibrils, increasing the matrix:mineral ratio. However, how the mechanisms of T1DM with HBO therapy affect the organic matrix is unknown.

Therefore, the present study suggested that fracture risk was increased in STZ-induced diabetic rats due to the reduced bone strength, energy and stiffness characterized by changes in collagen crosslinks [6, 27]. Our findings confirmed those of previous studies and increased the knowledge of how the mechanisms of HBO increase the stability of enzymatic crosslinks and may change organic and mineral bone matrix.

## Conclusion

The results showed that diabetes decreased collagen maturation and the mineral deposition process, reducing the bone capacity to absorb energy, maximum strength and stiffness. Moreover, the study showed that HBO improved the crosslink maturation and increased maximum strength and stiffness in the femur of animals with STZ-induced diabetes.
